# Microdosimetric Simulation of Gold-Nanoparticle-Enhanced Radiotherapy

**DOI:** 10.3390/ijms25179525

**Published:** 2024-09-02

**Authors:** Maxim Azarkin, Martin Kirakosyan, Vladimir Ryabov

**Affiliations:** P.N. Lebedev Physical Institute, 119991 Moscow, Russia; kirakosyanmr@lebedev.ru (M.K.); ryabov@lebedev.ru (V.R.)

**Keywords:** gold nanoparticles, MC simulation, microdosimetry, radiosensitization mechanisms

## Abstract

Conventional X-ray therapy (XRT) is commonly applied to suppress cancerous tumors; however, it often inflicts collateral damage to nearby healthy tissue. In order to provide a better conformity of the dose distribution in the irradiated tumor, proton therapy (PT) is increasingly being used to treat solid tumors. Furthermore, radiosensitization with gold nanoparticles (GNPs) has been extensively studied to increase the therapeutic ratio. The mechanism of radiosensitization is assumed to be connected to an enhancement of the absorbed dose due to huge photoelectric cross-sections with gold. Nevertheless, numerous theoretical studies, mostly based on Monte Carlo (MC) simulations, did not provide a consistent and thorough picture of dose enhancement and, therefore, the radiosensitization effect. Radiosensitization by nanoparticles in PT is even less studied than in XRT. Therefore, we investigate the physics picture of GNP-enhanced RT using an MC simulation with Geant4 equipped with the most recent physics models, taking into account a wide range of physics processes relevant for realistic PT and XRT. Namely, we measured dose enhancement factors in the vicinity of GNP, with diameters ranging from 10 nm to 80 nm. The dose enhancement in the vicinity of GNP reaches high values for XRT, while it is very modest for PT. The macroscopic dose enhancement factors for realistic therapeutic GNP concentrations are rather low for all RT scenarios; therefore, other physico-chemical and biological mechanisms should be additionally invoked for an explanation of the radiosensitization effect observed in many experiments.

## 1. Introduction

Radiotherapy (RT) is an effective and widely used cancer treatment modality. Nevertheless, RT often inflicts collateral damage to nearby healthy tissue. A better targeting of tumors by RT is a compelling task for medical physicists. Modern RT achieved significant success using highly sophisticated apparatus to focus the radiation field to tumors for various beam types. The advantage of the usage of proton and ion beams is a better targeting into the tumor over X-ray radiotherapy (XRT) due to a significant increase in deposited energy at the very end of the proton track, i.e., a well-known Bragg peak. In order to further increase therapeutic ratio, various radiosensitizers have been increasingly studied since the 1960s [1,2]. A quite novel approach to this problem is the usage of radiosensitizing nanoparticles [3], i.e., particles with sizes varying from few nms to ≈100 nm. Such particles generally have increased ability to penetrate through blood vessels to surrounding tissue, and show high cellular uptake [4]. In particular, nanoparticles consisting of high-*Z* elements are considered as dose enhancers due to huge photoelectric cross-sections at low photon energies that are proportional to $Z^{3}$–$Z^{5}$. The photoelectric processes can be followed by Auger electron emission. Produced electrons are soft (energy up to a few tens of keV), and have short absorption lengths in tissue that lead to a significant increase in the local dose deposition and ionization. Among heavy nanoparticles, gold nanoparticles are most widely studied due to their unique physico-chemical properties. Especially important for therapy are the following properties: gold nanoparticles are easy to prepare, have controllable shape and size, allow easier surface binding for functionalization, and have very high biocompatibility. Numerous studies demonstrate the increase in the effective absorbed dose in XRT with GNP both in vitro [5,6,7] and in vivo [8,9].

Recently, the idea to also use radiosensitizing nanoparticles was extended to proton therapy (PT) and other types of nanoparticles with different radiosensitizing mechanisms [10,11,12,13,14,15,16]. Enhancement of PT with nanoparticles is especially tempting, since it has the potential to dramatically decrease the damage to healthy tissue with respect to conventional XRT. The physics picture of proton beam interaction with tissue is different from XRT in many ways. Unlike XRT, irradiation by protons can induce nuclear reactions, leading to the production of neutrons, α-particles, unstable isotopes, and other products. Produced α-particles are of special interest, since they have high linear energy transfer (LET) and, therefore, relative biological effectiveness (RBE). For that reason, a number of elements were suggested to enhance production of α-particles in tumors for PT [14,17,18]. However, recent in silico studies show that the enhancement of α-particle production is negligible at realistic therapeutic concentrations of boron-11, which has the largest proton fusion cross-section of α-particle production [19,20,21]. Electromagnetic ionization of protons with matter mostly depends on the density of the medium, rather than *Z* of its atoms, which makes the advantage of heavy nanoparticles less evident. Nevertheless, in vitro [10,22,23,24,25] and in vivo [26,27] experiments show a significant radiosensitization effect in GNP-enhanced PT.

Contrary to nuclear processes [21], typical mean free paths of produced particles in electromagnetic interactions are at nanoscale, which is much less than the average distance between nanoparticles for therapeutic concentrations of GNPs. For that reason, an increase in dose deposition in the proximity of GNP can be one of the key effects relevant for the explanation of radiosensitization. Thus, in order to have a better picture of underlying mechanisms of the observed radiosensitizing effect induced by metallic nanoparticles under irradiation (XRT and PT), various studies based on Monte Carlo simulations have been undertaken [17,28,29,30,31,32]. These investigations were mainly focused on simulations of photons and proton interactions with tissue-like (mostly water) systems, with incorporated nanoparticles consisting of various materials at nanoscale. Thus, a significant increase in the dose in the proximity of a nanoparticle was found for both X-rays and proton therapy. Some studies went even further, and evaluated the production of reactive oxygen species around radiosensitizing nanoparticles [30,33]. However, current models of radiolysis are limited to production and propagation of a very few reactive species in pure water, which is substantially different in its nature from the complex chemical and biological properties of the cytoplasm of a living cell. The above-mentioned studies mostly consider simplified and idealistic systems where primary ionizing particles (beam) directly hit nanoparticles. There is a scarcity of full simulations that take into account the flux of secondary particles and realistic energy and spatial distribution of the incoming radiation field. A study of this kind was performed for XRT by Konefał et al. [34]. However, they did not use most recent discrete models of interactions of electrons in both water and gold, and simulated irradiation by high-energy protons only, i.e., 6 MV and 18 MV. One of the purposes of this work is to fill all of the above-mentioned gaps.

In this study, we simulate the interactions of proton beams, as well as 140 kVp and 6 MV X-rays, with a gold nanoparticles (GNPs) immersed in water using Monte Carlo simulation with Geant4 11.2.1 [35]. The advantage of this study is the usage of high-precision discrete models available in Geant4. The simulation in water is performed using microscopic physical models for calculations of biological damage induced by ionizing radiation at the DNA scale using Geant4-DNA [36,37]. To simulate the interactions of secondary electrons in GNPs, we use the novel microscopic Geant4_DNA_Au model [31,32]. The interaction of X-rays or proton beams with GNP and surrounding tissue is characterized with the dose enhancement factor (DEF) with respect to non-enhanced RT. In particular, we measure DEF as a function of a distance from the GNP center at microscopic scale. Also we calculate the macroscopic dose enhancement. Thanks to having the microscopic picture of a dose distribution, we are able to distinguish the energy deposited outside of GNP from the energy deposited inside a GNP. This approach has a significant impact on the macroscopic DEF in the living tissue, and is applied for the first time.

This paper is organized as follows: results of the simulation are given in Section 2. Section 3 discusses the nature of the radiosensitizing effect in light of the obtained results, and establishes connections between the results and various experimental data on NP-enhanced therapy. The geometry of the simulated system, radiation fields, and physics models used for this study are presented in Section 4. Finally, Section 5 summarizes our findings.

## 2. Results

We simulate the irradiation of a cubic tissue-like system with a size of 20 cm by 140 kVp and 6 MV X-rays and protons with an energy of 95 MeV. The layer located at a depth from 5 cm to 7 cm represents cancerous tissue loaded with spherical GNPs. The simulation is performed for GNPs with diameters of 10 nm, 20 nm, 40 nm, 80 nm. Interaction of the GNPs with the 140 kVp and 6 MV X-rays is studied only at depth of 7 cm, since the photon energy spectra have minor changes in their shape with depth. The energy of the protons was adjusted so that the Bragg peak position was at 7 cm, i.e., at the distal part of the tumor layer. The proton energy spectrum in frontal and distal parts of a tumor are very different; therefore, both scenarios of interaction protons with the GNP were studied. It should be noted that the large system size allowed us to account for secondary particles that also interact with GNP. A detailed description of the geometry of simulated system, radiation fields, and physics models used are given in Section 4. In the study, we measure spatial energy density enhancement factor ($DEF_{\mathrm{SE}}$) as a function of the distance from the center of the GNPs, which is defined as follows:

(1) $$DEF{\left(r\right)}_{\mathrm{SE}}=\frac{U_{\mathrm{GNP}}\left(r\right)}{U_{\mathrm{WNP}}\left(r\right)}=\{\begin{array}{ll} \frac{\rho_{\mathrm{Au}}}{\rho_{H_{2}O}}\frac{D_{\mathrm{GNP}}\left(r\right)}{D_{\mathrm{WNP}}\left(r\right)}, & \mathrm{if}\;r\leq R_{\mathrm{GNP}},\\ \frac{D_{\mathrm{GNP}}\left(r\right)}{D_{\mathrm{WNP}}\left(r\right)}, & \mathrm{if}\;r>R_{\mathrm{GNP}}. \end{array}$$

where $U_{\mathrm{GNP}}\left(r\right)$ and $D_{\mathrm{GNP}}\left(r\right)$ are the spatial density of the deposited energy and a dose as functions of a distance (*r*) from the GNP center, respectively; $U_{\mathrm{WNP}}\left(r\right)$ and $D_{\mathrm{WNP}}\left(r\right)$ are the spatial density of the deposited energy and dose as functions of the distance from the water nanoparticle (WNP) center, which has the size of the GNP and is placed at the same position; $R_{\mathrm{GNP}}$ is the GNP radius; and $\rho_{\mathrm{Au}}$ and $\rho_{H_{2}O}$ are the densities of gold and water, respectively. Both $U_{\mathrm{GNP}}\left(r\right)$ and $U_{\mathrm{WNP}}\left(r\right)$ are measured for the same fluxes of incoming radiation. Thus, $DEF{\left(r\right)}_{\mathrm{SE}}$ equals the conventional dose enhancement factor in the water region, whereas it gives a correct picture of the energy deposition inside a GNP.

**Figure 1:**
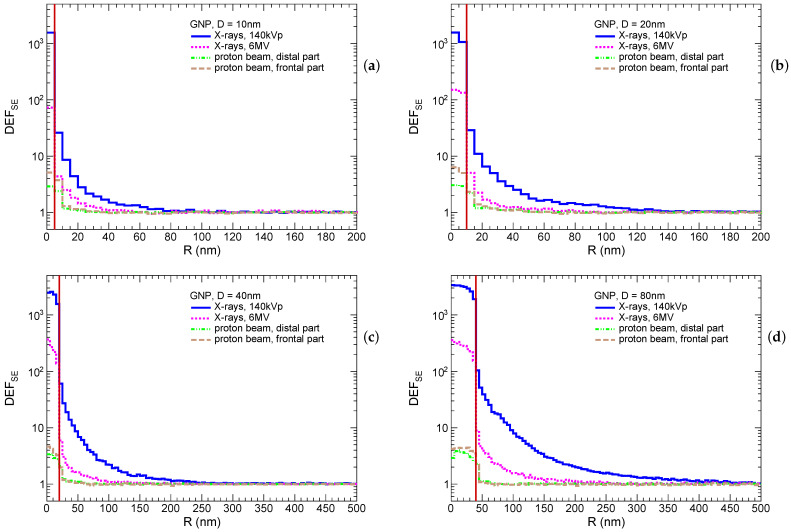
Spatial energy density enhancement factor ($DEF_{\mathrm{SE}}$) as a function of distance from the center of the gold nanoparticle (GNP) immersed in a homogeneous water system. The $DEF_{\mathrm{SE}}$s are measured for GNPs with diameters of 10 nm (**a**), 20 nm (**b**), 40 nm (**c**), and 80 nm (**d**). The vertical red line marks the GNP surface.

These distributions are shown in Figure 1. The most prominent $DEF_{\mathrm{SE}}$ is, as expected, observed for irradiation by 140 kVp X-rays for all particle sizes. In that case, the $DEF_{\mathrm{SE}}$ inside the GNP reaches ≈$2\times 10^{3}\text{–}3\times 10^{3}$, while it drops to ≈$2\times 10^{1}$–$10^{2}$ in the first 5 nm scoring shell outside the GNP, and then exponentially falls down, reaching the pedestal. The enhancement increases with the GNP size. For instance, the enhancement is indistinguishable from the pedestal (difference is below few percent) at 80 nm, 150 nm, 250 nm, and 450 nm for GNPs with sizes of 10 nm, 20 nm, 40 nm, and 80 nm, respectively. Similar observations are qualitatively applied to the irradiation by 6 MV X-rays; however, dose enhancements in first shell around GNP are lower, by a factor of ≈10, in comparison to the irradiation with 140 kVp X-rays. The radii of enhanced dose zones are reduced to 50 nm, 80 nm, 150 nm, and 250 nm for GNPs with sizes of 10 nm, 20 nm, 40 nm, and 80 nm, respectively. In the case of irradiation by protons, the enhancement reaches a factor of 3 to 6 inside the GNP, and ≈2 in the first 5 nm shell outside the GNP, and is negligible farther away. The enhancement is higher for a smaller GNP that can be explained by the relatively higher yield of soft secondary electrons accompanying protons inside a smaller GNPs to the contrary of a large one. The enhancement is also higher in the frontal part of the tumor, since more energetic protons with higher probability interact with inner electrons of gold atoms. It should be noted that the physics models of proton interaction with gold are not discrete. Hence, the production of δ-electrons inside GNPs may be distorted if their energies are comparable or lower than the average ionization potential (i.e., 790 eV for gold). However, such soft electrons have very short absorption lengths, and may affect the dose deposition only within few nanometers outside of the GNP.

It should be noted that bare GNPs are mostly used for in vitro experiments, while in vivo experiments tend to use decorated GNPs for functionalization. Most often, GNPs are coated by polyethylene-glycol (PEG) or polyacreylic acid to achieve a better stability and biocompatibility. Furthermore, other ligands can be attached to the coated GNP to facilitate a selective delivery to the tumor. Thus, the decoration can increase the diameter of nanoparticles by up to another 20 nm. Given the fact that all $DEF_{\mathrm{SE}}$s fall very steeply, the physical dose deposited in the living parts of cell is substantially reduced. This is especially important for GNP-enhanced PT, in which the dose enhancement is only observed in a few nanometers outside of the GNP. Properties of the decoration can also be changed by a significantly increased ionization near the surface of a GNP, but this goes beyond the scope of this study.

The simulated dose enhancement in the vicinity of a GNP looks quite large for X-rays. Hence, it is instructive to assess the total dose enhancement due to GNP at macroscopic scale. Most in vitro and in vivo studies of the radiosensitization effect use concentrations of gold ranging from few mg/L to 100 mg/L [38]. In our study, we set it to 10 mg/L. This concentration gives an average distance between GNPs of approximately 1 μm, 2 μm, 4 μm, and 8 μm for GNP sizes of 10 nm, 20 nm, 40 nm, and 80 nm, respectively. Such a sparse distribution of GNPs means that most of tissue between them is not exposed to GNP-induced radiation. It should be noted that the enhancement is present for X-rays, since the atoms of gold intercept some additional fraction of photons. The macroscopic dose enhancement is practically irrelevant for the proton beam, since almost all protons are supposed to lose all their energy before the distal side of the tumor anyway. The majority of studies of this kind evaluate macroscopic dose enhancement by integrating over entire volume, i.e., tissues and GNPs. In this study, using the microscopic picture of dose distribution, we assessed the macroscopic dose enhancement in the living tissue, excluding the GNP volumes. The approach is more relevant for the evaluation of the dose enhancement effect on physico-chemical and biological processes occurring in cells under irradiation. To quantify the dose enchantment at macroscopic scale, we use an additional relative dose (ARD), which is defined as follows:

(2) $$ADR=\frac{D_{\mathrm{GNP}}-D_{W}}{D_{W}},$$

where $D_{W}$ is a dose deposited in pure water (i.e., non-enhanced RT) and $D_{\mathrm{GNP}}$ is a dose received in RT enhanced with GNPs. The quantity is linear on concentration. The quantity measured in the volume excluding the GNPs is denoted as $ADR_{\mathrm{LT}}$, where “LT” stands for “living tissue”. The $ADR_{\mathrm{LT}}$ for different GNP sizes and radiation types are shown in Table 1. One can see that the $ADR_{\mathrm{LT}}$ decreases with the GNP size, which can be explained by higher energy absorption for larger nanoparticles. For the sake of completeness, we also measured the percentage of $ADR_{LT}$ in total ADR. It decreases with GNP size from approximately 25% to 15%. The absorption effect leads to the dependency of $ADR_{\mathrm{LT}}$ on the particular spatial distribution of nanoparticles in cell. It varies from a sparsely scattered one to a clustered one. For the latter, energy absorption inside nanoparticles may prove to be significantly higher if the average distance between nanoparticles would be of the order of hundred nanometers or less. It is worth mentioning that, as experimental studies show, nanoparticles are often very densely packed in clusters inside lysosomes [39].

## 3. Discussion

The macroscopic dose enhancement is small or even negligible for realistic therapeutic concentrations of gold nanoparticles for all kinds of RT. Dose enhancement in the vicinity of GNP is quite high for X-rays in the kilovoltage energy range, and still significant for megavoltage X-rays. By contrast, proton therapy demonstrates a very modest enhancement observed in the very close proximity of the GNP. Nevertheless, in vitro [22,23,24] and in vivo [26] experiments show a significant radiosensitization effect in GNP-enhanced PT. Moreover, elements with low and moderate *Z* also proved to be effective at radiosensitizing PT. For instance, nanoparticles consisting of TiO_2_, ZnO, Sodium Mercaptododecaborate, etc., have shown a significant radiosensitizing effect [14,40,41]. All of these facts indicate that multiple mechanisms should be involved.

It is generally accepted that RT results in an increased production of reactive oxygen species (ROS), causing oxidative stress and triggering apoptosis. A number of recent studies of GNP (and some other NP)-enhanced radiotherapy strongly indicates an increased production of ROS with respect to the non-enhanced one. Physico-chemical GNP-related mechanisms that facilitate the increase in ROS generation in cells are GNP-enhanced radiolysis and radiation-induced catalytic enhancement of ROS production. The former is proportional to dose enhancement, at least according to the known mechanisms of radiolysis [33], and proved to be small on macroscopic scales, whereas the latter ought to have a huge enhancement factor to be viable of causing observed radiosensitization. It is established that the electrically active surface of GNPs and their high surface-to-volume ratio may provide catalyzation of chemical reactions [42]. Ionzing radiation is assumed to enhance the catalytic property of ROS generation by production of additional donor electrons [43]. Low work function can further facilitate these processes [44]. It should be noted that radiation-enhanced catalysis takes effect during the physical stage of interaction of ionizing radiation with nanoparticles at very short time scales. Unfortunately, as far as the authors are concerned, there are still neither theoretical nor MC-based quantitative estimations of the radiation-induced catalytic enhancement factor based on first principles. It is worth mentioning that the catalytic properties may also be tightly conjugated with the functionalization [45], making the process of ROS production even more complex. Thus, it is still not clear whether catalysis may cause few orders of enhancement needed for the explanation of observed sensitization by GNPs.

Another radiosensitization mechanism may be connected with the acquisition of a positive charge by NP via ionization caused by radiation [46,47]. It is generally accepted that positively charged NPs have higher cellular uptake and cytotoxicity than neutral or negative ones [38,48,49,50]. It was found that positively charged GNPs can cause oxidative stress, interfere with the cell signaling system, and inhibit DNA reparation [49,50]. The observations show that most kinds of NPs have surface charges from −30 mV to +30 mV, whereas NPs with surface charges of >20 mV demonstrate an acute cytotoxic effect [45,51]. The long-range displacement of electrons from the nanoparticle by irradiation can significantly alter potential at its surface, making it more cytotoxic. For instance, the potential of a spherical system outside of the sphere is given by the formula:

(3) $$\phi \left(r\right)=\frac{Q}{4\pi \varepsilon_{0}r},$$

where *Q* is a total charge of the system, *r* is a distance from the center of the system, $\varepsilon_{0}$ is the vacuum electric permittivity. For example, a single displaced electron from a GNP with the size of 20 nm can add the following electric potential at its surface: $\phi \left(20\;\mathrm{nm}\right)=1.6\times 10^{-19}\;C/\left(4\pi \cdot 8.9\times 10^{-12}\;F\cdot m^{-1}\cdot 20\times 10^{-9}\;m\right)\approx$ 75 mV. Obviously, the electron yield from the surface of a nanoparticle goes in parallel with the opposite process of the capture of free electrons from the medium because of Coulomb attraction between a positively charged nanoparticle and free electrons in the medium. In the end, the kinetic interplay between these two processes and their time scales determines the resulting electric charges of nanoparticles in media and their dependence on time. The above-mentioned kinetic is not sufficiently investigated at the moment for different types of nanoparticles neither experimentally nor theoretically. Nevertheless, one can cautiously assume that the acquired charge may be non-negligible on macroscopic time scales in cell environment.

An effect of this kind was observed for TiO_2_ nanoparticles irradiated UV light [52,53]. Photoexcited electrons were emitted from nanoparticles to surrounding bulk and increased Z-potential of the nanoparticle. It is of special importance that the change in surface charge was persistent over time. The persistence of increased charge of the surface came up in in vitro experiments [53,54]. Thus, nanoparticles pre-exposed to UV light decreased cell viability in comparison to pristine nanoparticles. One should note these studies reported that pre-exposed nanoparticles did not increase ROS level, and did not cause an oxidative stress.

The above-discussed physical and physico-chemical mechanisms are followed by various biological processes. It is argued that sensitization to radiation can happen due to biological mechanism triggered by GNP prior to irradiation. For instance, it was found that GNPs can bind endogenous antioxidants inside cells, making them vulnerable to radiation. Moreover, it was shown that GNPs cause ROS production via inhibition of thioredoxin reductase 1 and other redox-relevant mechanisms (see [55] and the references therein). While these effects are beyond the scope of this study, it is, however, natural to assume that biological GNP-induced and radiation-induced processes can be synergistic in NP-enhanced RT.

All in all, large photoelectric cross-sections of gold result in hugely increased ionization in very close proximity to the GNPs, and may serve as an initial seed to increase the charge of the nanoparticles in XRT, especially at kilovoltage energies. The interactions of protons with atoms of gold results in an insignificant increase in the local dose deposition; therefore, the mechanisms of the observed radiosensitization are connected to the soft energy physico-chemical processes. In conclusion, various physico-chemical (e.g., catalysis of ROS generation) and biological mechanisms ought to be invoked for the explanation of the observed radiosensitization effect in all kinds of RT.

## 4. Methods and Models

### 4.1. Geometry of the Simulated System

**Figure 2:**
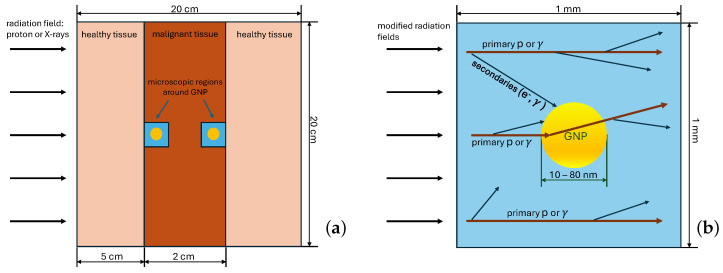
A schematic (not to scale) layout of the simulated system: (**a**) The macroscopic setup represents a cube consisting of human soft tissue. The dark orange layer represents a tumor, the smaller blue cubes represent the microscopic volume with a gold nanoparticle inside, either on frontal or distal parts of the tumor layer. (**b**) A close-up of a microscopic volume represents a water cube with gold nanoparticles of various (10 to 80 nm) radii inside. The thick dark red arrows denote the primary beam particles, and the thin black arrows indicate the secondary particles that can interact with the GNP as well.

The simulated system is represented a the cube with a side of 200 mm filled with a simplistic tissue-like material, which can be accessed in Geant4 11 by calling the class G4HumanPhantomMaterial with the argument “*soft_tissue*”. The material consists of a simple mixture of elements, comprising human soft tissue and having the density of human soft tissue. The proton beam or X-rays cross the cube perpendicularly to one of its faces, which is called frontal one. The layer located on depths between 50 mm and 70 mm with respect to the frontal face, represents a malignant tumor. This layer contains a microscopic cubic water volume with a size of 1 mm placed at either frontal or distal sides of the tumor. The microscopic volume has a GNP in its center. We study the interaction of radiation with GNPs with diameters of 10 nm, 20 nm, 40 nm and 80 nm. Two cases of different microscopic volume positions (either at the frontal or distal sides of the tumor) are considered for PT, since the proton energy significantly changes in the last 2 cm of its track (see Section 4.2). Differences in the energy spectra of X-rays at the frontal and distal sides are minor; therefore, only the distal side is studied in the case of XRT. A layout of the simulated system is shown in Figure 2.

To conduct the study using reasonable computing resources, macroscopic and microscopic simulations of the beam interactions are performed in two stages. In the first stage, the proton beam and X-rays are passed through a thick layer of tissue in the geometric setting described above. This provides the energy spectrum of the primary beam particles in the tumor region for the second stage of the simulation. In the second stage, the modified beams interact with a microscopic system represented by a cube with a side of 1 mm, with a GNP placed in its center. The size of the microscopic system is chosen so that most of secondary particles relevant for microdosimetric studies would be accounted for; the 1 mm size is more than enough, since the typical energy of secondaries is, at most, of an order of ∼ keVs and they have absorption lengths of no more than a few micrometers. The simulation of a beam transport through the macroscopic tissue layer is performed using condensed history physics models, while the simulation of an irradiation of the microscopic region is performed using physics models dedicated for microdosimetric studies. Both approaches are described in detail in Section 4.3.

### 4.2. Radiation Fields

**Figure 3:**
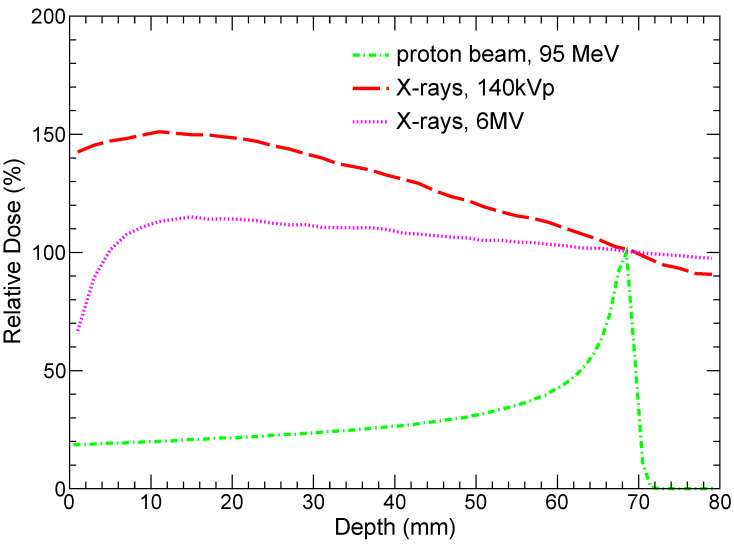
Relative dose versus tissue depth for different types of radiation.

The system is irradiated with a uniform flux of photons or protons. The energy spectra of the incoming beam particles are chosen so that they would be close to realistic RT scenarios. Namely, we study an irradiation by X-rays with a 140 kVp spectrum [56], 6 MV photon beam [57], and a proton beam with an energy of 95 MeV. The chosen X-ray spectra are typical for modern CT and XRT machines. The energy of the protons is adjusted so that Bragg peak is close to the distal part of the tumor layer that is demonstrated in Figure 3, which shows the relative dose versus depth in tissue for both protons and X-rays. It is instructive to note that the doses deposited by both kilovoltage and megavoltage X-ray beams monotonically decrease from a depth of ≈10 mm, and are undesirably high outside of the tumor slice.

**Figure 4:**
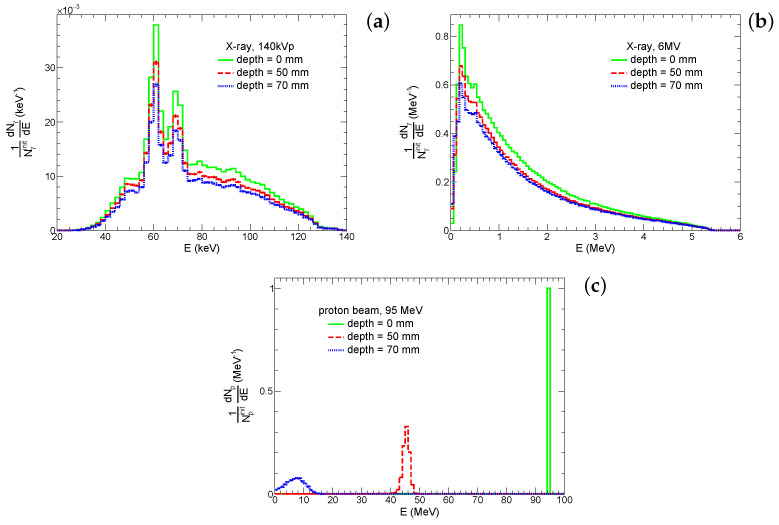
Energy distribution of 140 kVp (**a**), 6 MV (**b**), and photons and protons (**c**) at different depths in tissue. These distributions are normalized by the number of initial beam photons ($N_{\gamma }^{\mathrm{init}}$) and protons ($N_{p}^{\mathrm{init}}$).

The energy distributions of photons and protons at the frontal side of the system and edges of the tumor layer for the corresponding beams are shown in Figure 4. These spectra of protons and photons at the frontal and distal sides of the tumor are used for irradiation of the microscopic system at the second simulation stage (see Section 4.3). One can see that energies of protons at depths of 5 cm and 7 cm are ≈45 MeV and of the order of few MeVs, respectively. Therefore, the nature of proton interactions with tissue and the incorporated GNPs is expected to be different, and both scenarios are considered. Thus, for the sake of a clearer physical picture, we use both energy spectra for the simulation of irradiation of the microscopic system by protons. In a real PT, the tumor is exposed to the proton beam from different directions and with different energies for better conformity and coverage of the tumor. Thus, the irradiation of different tumor parts is limited by these two cases. Conversely, the shape of energy spectra of both kilovoltage and megavoltage photons gradually changes with the depth. The differences between the frontal and distal sides of the tumor layer are minor; therefore, we simulate the irradiation of the microscopic system by X-rays only in the distal part of the tumor layer. However, it should be noted that, for 6 MV X-rays, a fraction of the low-energy photons ($E_{\gamma }<$ 200 keV) significantly increases with depth. Such photons make a major contribution to the photoelectric effect for high-*Z* atoms. Therefore, the yield of the corresponding physics processes and, hence, therapeutic outcome can be different for the GNP-enhanced XRT of superficial and internal tumors.

### 4.3. Physics Models

The aim of this study is an in silico investigation of the patterns of the dose deposition in the vicinity of GNPs for PT and XRT. The simulations is performed using an open source package for the simulation of particle propagation in matter: Geant4 [35], version 11.2.1. Nowadays, Geant4 is widely used in various fields of physics, from high energy and cosmo-physics to medicine. In the Geant4 approach, the user defines the geometry, physical processes, and specific models for the interaction of interested particles with a given media, while Geant4 Monte Carlo algorithms perform the simulation. The object-oriented nature of Geant4 allows users to choose between models for different physical processes at various energy ranges. A wide range of physics models have been implemented in decades of development. Usually, a certain set of physical models is prepackaged to be used for particular simulation purposes. These sets are called physics lists. As mentioned in Section 4.1, our simulation setup has macroscopic and microscopic stages, both using dedicated physics models.

Let us briefly describe the physics models used in our Geant4 setup. The propagation of photon and proton beams in the macroscopic tissue layer is performed with the QBBC physics list. For electromagnetic physics, QBBC uses a standard constructor G4EmStandardPhysics_option0. The QBBC physics list also includes hadronic models for simulations of hadronic processes in tissue. The output spectra of the primary beam particles from the macroscopic stage are used as inputs for the next microscopic stage of the simulation. Thus, we simulate the microscopic transportation of beam particles (namely protons or photons) and all secondaries through the microscopic volume, consisting of water, with a gold nanoparticle in its center. It should be noted that water was chosen because the most advanced microscopic models in Geant4 are available only for water among all other tissue-like materials. The particle transport inside the microscopic volume is performed with microscopic or discrete models in Geant4 with maximum precision.

Namely, Livermore models are used for the photon transport, i.e., the photoelectric effect, gamma conversion, and Compton and Reyleigh scattering. For the proton and electron transport in water, the Geant4-DNA set of models are used, while in gold, a recently implemented set of models for microscopic electron transport [31,32] were utilized. Unfortunately, at the moment, discrete models for proton interactions in gold are not available. Thus, for proton transportation inside gold nanoparticles, standard Geant4 models have been implemented for proton ionization, bremsstrahlung, electron–positron pair production, and elastic scatterings. Since the Geant4 models for proton interactions in gold are not discrete, they may distort the energy loss of beam particles and production of low-energy secondaries. However, for lack of a better option, we use the standard Geant4 models for proton interactions with gold. The atomic de-exitation processes (Auger electrons, Auger cascade, particle-induced X-ray emission, and fluorescence) are accounted for by the use of the Geant4 G4UAtomicDeexcitation methods. The production cuts for photons, electrons, and protons were chosen to be 0.1 nm (low edge parameter—1 eV). To switch between models in different Geant4 regions, the “Sakata method” [31,32] from Geant4 example/extended/medical/dna/AuNP was implemented.

More elaborately, to account for proton or photon interactions with tissue on the macroscopic scale (see Figure 2a), the QBBC physics list is used. The spectra of protons and photons after passing the thick layer (5 to 7 cm) of human soft tissue are used as an input for the main simulation code at the nanoscale. The geometry at this stage consists of two microscopic regions, as represented on Figure 2b:

Region (1): the 1 millimeter cube of water to account for production of secondaries.
Region (2): gold nanoparticles of various radii inside the cell.

In these microscopic regions, the Livermore models were used for photon–gold interactions. For electrons and protons, physics models depend on the region. Specifically, the following set of models was used for electrons:

- In the water region, Geant4-DNA models were implemented.
- In the GNP region, a new set of Geant4-DNA discrete models for electron interaction with gold were implemented [31].

For protons:

- In the water region, Geant4-DNA models were implemented.
- In the GNP region, standard Geant4 models were used for lack of a better option.

## 5. Conclusions

In this study, we obtained the dose enhancement factors in tissue in the vicinity of GNPs using Monte Carlo simulations with Geant4 11.2.1, and most recent discrete models of particle tracking in liquid water and gold for three types of radiation: proton beams, and kilovoltage and megavoltage X-rays. The dose enhancement factors were measured for spherical GNPs with diameters of 10 nm, 20 nm, 40 nm, and 80 nm. It is worth mentioning that the simulation took in into account the effects of the beam passage to the tumor through thick tissue layers, including the interactions of secondary particles with a GNP.

The most prominent dose enhancement is observed for 140 kVp X-rays. Thus, the dose enhancement factor in the first 5 nm shell outside of the GNP increases with its size from ≈$3\times 10^{1}$ to ≈$10^{2}$, whereas it falls down close to unity at distances of 80 nm, 150 nm, 250 nm, and 450 nm for GNPs with sizes of 10 nm, 20 nm, 40 nm, and 80 nm, respectively. The shape of $DEF_{\mathrm{SE}}$ profiles for 6 MV X-Rays are close 140 kVp X-rays; however, the values of $DEF_{\mathrm{SE}}$s are one order lower compared to 140 kVp X-rays, and dose enhancement zones are nearly two times smaller for all GNP sizes. The dose enhancement factor for proton beam is ≈2 in the first 5 nm shell outside of a GNP, whereas it is negligible further away from the GNP surface.

The dose enhancement in the proximity of GNP for XRT turns out to be very high, while the macroscopic dose enhancements are still negligible for realistic therapeutic concentrations of GNPs in tissue. Therefore, modification of the surface charge and other physico-chemical properties of the GNPs ought to play a major role in the sensitization process. The increased level of ROS is considered to be one of the main reasons for the decrease in the survival of cells exposed to radiation. The yield of produced ROS is expected to be proportional to a dose enhancement factor. Given a small dose enhancement for all types of radiation at realistic GNP concentrations, it becomes clear that other radiation-induced mechanisms of ROS generation should be involved. Hence, enhanced ROS production can be connected to a nanoparticle-mediated biological redox processes. It should be noted that there is evidence that NPs can reduce cell survival without enhanced ROS production, suggesting that other biochemical mechanisms may come into play. The radiosensitization in PT almost certainly caused neither local dose enchantment, nor related ROS production in the physical stage of irradiation, and the biological processes should play a major role here.

## Figures and Tables

**Table 1:** Additional relative dose ($ARD_{\mathrm{LT}}$) delivered to the tumor due to the presence of GNP of different sizes at macroscopic scale. The dose is scored excluding GNP volume. The ARD is measured for GNP concentration in tissue of 10 mg/L.

Radiation Type	GNP Size			
	10 nm	20 nm	40 nm	80 nm
X-rays, 140 kVp	$2.4\times 10^{-4}$	$2.1\times 10^{-4}$	$1.9\times 10^{-4}$	$1.6\times 10^{-4}$
X-rays, 6 MV	$3.5\times 10^{-5}$	$2.7\times 10^{-5}$	$2.3\times 10^{-5}$	$1.8\times 10^{-5}$
proton beam	not applicable			

## Data Availability

The code used for simulations presented in this study is available upon request to authors.
